# The Characteristic Aroma Compounds of GABA Sun-Dried Green Tea and Raw Pu-Erh Tea Determined by Headspace Solid-Phase Microextraction Gas Chromatography–Mass Spectrometry and Relative Odor Activity Value

**DOI:** 10.3390/foods12244512

**Published:** 2023-12-18

**Authors:** Chenyang Ma, Chang Gao, Yuanda Li, Xiaohui Zhou, Guofu Fan, Di Tian, Yuan Huang, Yali Li, Hongjie Zhou

**Affiliations:** 1 College of Tea Science, Yunnan Agricultural University, Kunming 650500, China; machenyang0305@icloud.com (C.M.); 15825226653@163.com (C.G.); 18887449386@163.com (X.Z.); f1179643966@163.com (G.F.); 2 Laboratory of Tea Science of Ministry of Education, Hunan Agricultural University, Changsha 410128, China; 13398802207@163.com; 3 College of Food Science and Technology, Yunnan Agricultural University, Kunming 650500, China; m17853435722@163.com; 4 College of Pu-Erh Tea, West Yunnan University of Applied Sciences, Puer 671000, China; 17787107713@139.com

**Keywords:** GABA sun-dried green tea, GABA raw Pu-erh tea, characteristic aroma compounds, metabolic pathways, relative odor activity

## Abstract

We aim to improve the product quality of GABA raw Pu-erh tea during development and processing. In this study, headspace solid-phase microextraction gas chromatography–mass spectrometry technology combined with relative odor activity evaluations was used to compare the volatile compounds of GABA sun-dried green tea and GABA raw Pu-erh tea. Sensory evaluation showed a higher aroma score of GABA raw Pu-erh tea than that of GABA sun-dried green tea, with significant differences in aroma type and purity. A total of 147 volatile compounds of 13 categories were detected, which differed in composition and quantity between the two teas. 2-Buten-1-one,1-(2,6,6-trimethyl-1,3-cyclohexadien-1-yl)-,(E)- and beta.-myrcene largely contributed to the aroma formation of both teas. Five volatile compounds were screened as potential markers for tea aroma. Metabolic pathway analysis showed that monoterpenoid biosynthesis may be beneficial to the formation of flowery and fruity aromas in the teas. We suggest that the findings of this study may provide important guidance for the processing and optimization of GABA tea.

## 1. Introduction

Yunnan, China, is the origin center of the tea tree, contributing to a culture characterized by tea drinking for more than 1000 years [1]. Pu-erh tea is a traditional and popular type of tea with a long history in Yunnan. According to the processing type and quality characteristics, Pu-erh tea can be divided into raw and ripe forms [2]. In recent years, Pu-erh tea has become increasingly popular with consumers because of its unique health benefits and quality characteristics [3]. Raw Pu-erh tea is a tightly pressed tea that is prepared by steaming and drying Yunnan big-leaf sun-dried green tea. Raw Pu-erh tea has the quality characteristics of a pure and lasting aroma, green and yellow clear soup color, and strong and sweet taste [4]. The aroma type of raw Pu-erh tea is mainly clean and refreshing, accompanied by nutty, grassy, and fatty aromas. The composition of volatile substances is similar to that of sun-dried green tea, albeit with some distinct differences [5,6]. After dry storage, the aroma of raw Pu-erh tea gradually changes to a woody, sweet, and unique aging aroma [7]. With the goal of developing raw Pu-erh tea products with unique flavor, many recent studies have explored the influence of traditional processing technology on the flavor of the tea. Fan et al. collected samples at different processing stages for determination of volatile compounds (VCs), showing that deactivation and autoclaving were the key processing steps to form the flavor substances of raw Pu-erh tea. Deactivation significantly increased the relative content of aldehydes and significantly reduced the relative content of olefins. Autoclaving further reduced the contents of aldehydes and esters and significantly increased the contents of nitrogen-containing compounds and ketones. Therefore, exploring appropriate processing parameters may offer new methods to regulate the flavor of raw Pu-erh tea [8]. Feng et al. found that the VCs in raw Pu-erh tea were mainly alcohols, terpenes, aldehydes, and ketones. Through aroma recombination experiments, it was determined that light, fat, flower, and fruit aromas were the characteristic aroma attributes of raw Pu-erh tea, providing a theoretical basis for processing different flavors of raw Pu-erh tea [9].

In 1987, Tsushida and colleagues discovered the method of gamma-aminobutyric acid (GABA) enrichment in tea, leading to the successful development of GABA teas (with a GABA content above 1.5 mg/g tea) [10,11]. Since the content of GABA in ordinary tea is generally low, nitrogen-filled anaerobic methods are usually used to enrich GABA in tea. Under anaerobic conditions, GABA in tea is mainly enriched by GABA shunt and polyamine degradation [12]. GABA tea has also been favored by the public because of its unique flavor and pharmacological effects. Zhen found that the flavor of GABA green tea processed by an anaerobic method changed significantly compared with that of ordinary green tea, resulting in an obvious sweet and sour fruit aroma, accompanied by increased contents of methylphenol, methyl myristate, methyl laurate, and methyl palmitate [13]. Li et al. found that GABA white tea processed by an anaerobic method exhibited a strong floral and fruity aroma, which was mainly due to 2-heptanol [14]. Our previous study demonstrated that the GABA content in the fresh leaves of Yunnan big-leaf tea trees can be effectively accumulated after 6 h of anaerobic treatment. The processed GABA sun-dried green tea and GABA raw Pu-erh tea products exhibited unique flavor qualities with certain promotion value [15].

To date, more than 700 VCs have been identified in tea, but only a small number of VCs have been confirmed to contribute to the formation of the characteristic tea flavor [16]. The formation of the characteristic flavor of tea is not only affected by the concentration and taste but is also the result of the interaction between the constituent compounds and is closely related to the olfactory threshold (OT) [17]. Therefore, determination of the odor activity value (OAV) and relative odor activity value (ROAV) is considered to be an important method for screening and forming characteristic aroma compounds [18,19]. In addition, headspace solid-phase microextraction (HS-SPME) technology, gas chromatography–mass spectrometry (GC-MS) technology, gas chromatography–olfactometry technology, electronic nose technology, sensory evaluation, Kyoto Encyclopedia of Genes and Genomes (KEGG) pathway analysis, aroma recombination and omission experiments, and other methods [20,21] are commonly used for the screening, identification, and testing of characteristic aroma compounds in food research. Some of these techniques have also been applied to the analysis of tea. For example, Hong et al. used HS-SPME-GC-MS technology combined with sensory evaluation results and then applied OAV and multivariate statistical analysis to explore the characteristic aroma compounds of Chinese yellow tea with different aroma types. They identified 8, 14, 7, and 18 VCs in yellow tea with flowery, high-fired, fresh, and corny aromas, respectively, which could be used as the characteristic aroma compounds of yellow tea with different aroma types [22].

In this study, sensory evaluation, HS-SPME-GC-MS combined with ROAV, and multivariate analysis were used to compare and analyze the VCs of GABA sun-dried green tea and GABA raw Pu-erh tea. The key VCs of aroma formation and the main metabolic pathways of biosynthesis in GABA sun-dried green tea and GABA raw Pu-erh tea were clarified. These results are expected to provide a theoretical basis for optimizing the processing technology of GABA sun-dried green tea and GABA raw Pu-erh tea.

## 2. Materials and Methods

### 2.1. Tea Samples

Four groups of samples, including fresh tea leaves as a control (CK), anaerobic-processed tea leaves (CA), GABA sun-dried green tea leaves (SGT), and GABA raw Pu-erh tea leaves (PRT), were prepared and compared. Three replicates were established for each group. In July 2021, all samples (one bud, two or three leaves of the same tea variety *Camellia assamica* vs. Yun kang 10) were picked and processed in the tea garden of Dalishu Tea Factory in Yunlong County, Dali, China.

**Figure 1:**
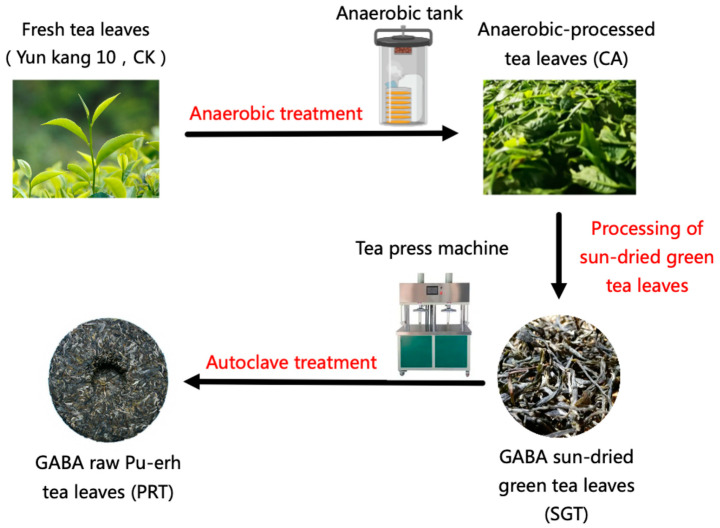
The process of GABA sun-dried green tea and GABA raw Pu-erh tea manufacturing.

In the CK group, the fresh leaves of tea plants were deactivated by steam at 100 °C for 5 min and then dried at 45 °C for 2 h. In the CA group, the fresh leaves of tea plants were placed in a special machine filled with N_2_ for anaerobic treatment for 6 h, vaporized at 100 °C for 5 min, and then dried at 45 °C for 2 h. In the SGT group, the fresh leaves of the tea plant were air-dried for 2 h and then placed in a special machine filled with N_2_ for anaerobic treatment for 6 h, followed by steam deactivation at 100 °C for 3 min, rolling for 45 min, and finally dried by sunshine for 8 h. In the PRT group, the fresh leaves of the tea plant were aired for 2 h, placed in a special machine filled with N_2_ for anaerobic treatment for 6 h, and then vaporized at 100 °C for 3 min. After rolling for 45 min, the tea leaves were dried by sunshine for 8 h, autoclaved into a cake type, cooled to room temperature, dried for 3 h, and finally dried at 60 °C for 1 h. The manufacturing procedures are specified in the Chinese national standard procedure and are shown in Figure 1.

### 2.2. Chemicals

N-hexane (chromatographical purity) was purchased from Merk (Darmstadt, Germany). NaCl (analytical purity) was obtained from China National Pharmaceutical Group Co., Ltd. (Kunming, China). C7-C40 (chromatographical purity) saturated alkanes and standards were purchased from Sigma-Aldrich (Darmstadt, Germany), which were used to determine linear retention indices.

### 2.3. Sensory Panel Evaluation of Teas

Sensory evaluation of tea samples was carried out according to Chinese national standard procedure [23]. The members of the tea review team were all from the Tea College of Yunnan Agricultural University. The team included 10 trained reviewers (five females and five males with an average age of 30 years and professional experience of 5–35 years) who evaluated the appearance and quality of tea samples according to the Chinese national standard procedure. The score was calculated by the method of percentage weighting. First, the tea evaluation terms were discussed, and then the sensory evaluation was carried out in the special tea review room at room temperature (25 °C). The tea samples were delivered in random order to ensure that the team members did not know the sample number [24]. Then, 100 g samples were randomly selected to be evaluated and have their appearance scored (A). Next, 3 g of each tea sample was taken and brewed with 150 mL of boiling water. After 5 min, the beverage was used to evaluate and score the internal quality, including aroma (B), beverage color (C), taste (D), and leaf base (E). Finally, the opinions of the group members were summarized, and the quality of tea samples was quantified according to the following formulas:

SGT review score = A × 25% + B × 25% + C × 10% + D × 30% + E × 10%

PRT review score = A × 20% + B × 30% + C × 10% + D × 35% + E × 5%

### 2.4. Sample Preparation

All sample preparation and detection methods were improved by referring to the methods in the existing papers [25]. Materials were harvested, weighed, immediately frozen in liquid nitrogen, and stored at –80 °C until needed. Samples were ground to a powder in liquid nitrogen. One gram of the powder was immediately transferred to a 20 mL headspace vial (Agilent, Palo Alto, CA, USA) containing NaCl saturated solution to inhibit any enzyme reaction. The vials were sealed using crimp-top caps with TFE-silicone headspace septa (Agilent). At the time of SPME analysis, each vial was placed in a 100 °C water bath for 5 min, and then a 120 µM divinylbenzene/carboxen/polydimethylsilioxan fiber (Agilent) was exposed to the headspace of the sample for 15 min at 100 °C.

### 2.5. GC-MS Conditions

After sampling, desorption of the VCs from the fiber coating was carried out in the injection port of the GC apparatus (Model 8890; Agilent) at 250 °C for 5 min in splitless mode. The identification and quantification of VCs were carried out using an Agilent Model 8890 GC and a 5977B mass spectrometer (Agilent), equipped with a 30 m × 0.25 mm × 0.25 μM DB-5MS (5% phenyl-polymethylsiloxane) capillary column. Helium was used as the carrier gas at a linear velocity of 1.2 mL/min. The injector temperature was kept at 250 °C, and the detector temperature was maintained at 280 °C. The oven temperature was programmed from 40 °C (3.5 min), increasing at 10 °C/min to 100 °C, at 7 °C/min to 180 °C, at 25 °C/min to 280 °C, and held for 5 min. Mass spectra were recorded in electron impact ionization mode at 70 eV. The quadrupole mass detector, ion source, and transfer line temperatures were, respectively, set at 150 °C, 230 °C, and 280 °C. Mass spectra were scanned in the m/z range of 50–500 amu at 1 s intervals. Identification of VCs was achieved by comparing the mass spectra with the data system library (MWGC or NIST) and linear retention index.

### 2.6. ROAV Calculation

Based on the relative content of aroma compounds and the threshold value of each aroma component in water, the ROAV calculation formula is determined as follows:

ROAV = (C_n_/C_max_) × (T_max_/T_n_) × 100

where C_n_ represents the relative content of any VC (%), C_max_ represents the relative content of the largest VC (%), T_n_ represents the OT of any VC, and T_max_ represents the maximum OT of VCs; the greater the ROAV value, the greater the contribution value of the compounds to the aroma. VCs with ROAV > 1 can be considered to have an important contribution to the composition of the aroma, whereas VCs with 0.1 < ROAV < 1 can be considered to have a modification effect on the composition of the aroma [26].

### 2.7. KEGG Pathway Analysis

To explore the biosynthesis pathways of aroma compounds during the processing of tea samples in the SGT and PRT groups, the detected VCs were compared with the known compounds in the KEGG database (https://www.kegg.jp/kegg/pathway.html (accessed on 15 August 2023)), and metabolic pathway analysis was performed using MBRole 2.0 (http://csbg.cnb.csic.es/mbrole2/ (accessed on 15 August 2023)) [27].

### 2.8. Statistical Analysis

All data were measured three times in parallel, and the results are expressed as mean ± standard deviation. SPSS v.26 was used for analysis of variance, and Tukey’s post-hoc test. *p* < 0.05 was used as the standard for judging a significant difference between groups. Metware Cloud was used for principal component analysis (PCA) and orthogonal partial least-squares discriminant analysis (OPLS-DA). Draw Venn and Origin 2022 were used for drawing the clustering heat map and other data analysis and mapping.

## 3. Results and Discussion

### 3.1. Sensory Evaluation of Aroma Characteristics of GABA Sun-Dried Green Tea and GABA Raw Pu-Erh Tea

The sensory evaluation method, as the most basic tea quality evaluation method, is based on people’s intuitive feelings. Nevertheless, this method remains irreplaceable and is widely used for the evaluation of all tea types [28,29]. The evaluation of aroma factors in tea sensory evaluation usually includes four aspects: aroma type, concentration, purity, and persistence [30].

As shown in Table 1, the aroma characteristics of the SGT and PRT were significantly different. The aroma of SGT was fragrant, high, pure, and lasting, and the flowery aroma was rich, while the fruity aroma was weak. The aroma of the PRT was purer and longer-lasting and had flowery, fruity, and sweet characteristics. The aroma factor score and total scores of PRT were higher than those of SGT. Since a previous study showed that the processing type (autoclaved) can enhance the flavor of raw Pu-erh tea [31], and the autoclaved process is a unique part of the PRT, the autoclaving process may be the cause of the difference in aroma characteristics between the two tea samples.

### 3.2. Quantitative and Qualitative Analysis of VCs in GABA Sun-Dried Green Tea and GABA Raw Pu-Erh Tea

**Figure 2:**
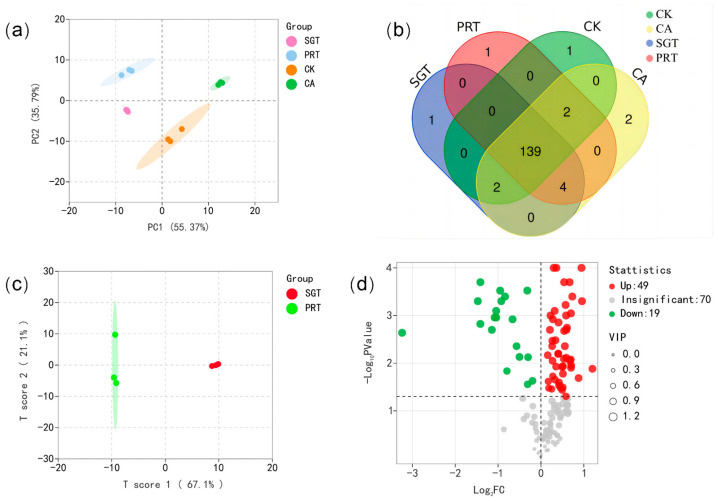
Multivariate statistical analysis of volatile organic compounds in the processing of GABA sun-dried green tea and GABA raw Pu-erh tea. (**a**) PCA model score scatter plot (total). (**b**) Venn diagram of volatile organic compounds. (**c**) Scatter plot of scores of OPLS-DA model (SGT vs. PRT). (**d**) Volcano plot of differential compounds (SGT vs. PRT). CK refers to fresh tea leaves, CA refers to anaerobic tea leaves, SGT refers to GABA sun-dried green tea, and PRT refers to GABA raw Pu-erh tea.

Using HS-SPME-GC-MS technology to detect GABA sun-dried green tea and GABA raw Pu-erh tea, a total of 148 VCs were identified (Table 2). Among them, 145, 145, and 143 VCs were detected in SGT, PRT, and CK samples, respectively, and all 148 VCs could be detected in CA samples. After comparing the types of VCs between tea samples, it was found that after anaerobic treatment of CK, a total of five VCs were newly generated, including 9,12-octadecadienoic acid, (Z,Z)-, methyl ester; 9,12,15-octadecatrienoic acid, (Z,Z,Z)-; (Z)-9,17-octadecadienal; linoleic acid ethyl ester; and (E)-2-hexenoic acid, butyl ester. The first four VCs were also detected in both SGT and PRT, and their contents increased. Therefore, further study is needed to determine whether these four compounds affect the formation of SGT and PRT aroma characteristics. However, the last VC was only detected in CA, indicating that anaerobic treatment can increase the type of VCs formed. After SGT was autoclaved into PRT, two new VCs were generated, 2-(1,1-dimethylethyl)-6-(1-methylethyl) phenol and 4H-pyran-4-one,2,3-dihydro-3,5-dihydroxy-6-methyl-. Further analysis showed that tea samples with different treatments could be clustered, indicating that the data were representative [32], and there were significant differences in the contents of VCs among different tea samples (Figure S1). The results of PCA showed that the four groups of tea samples could be clustered separately with obvious separation between the groups (Figure 2a). In addition, there were also some differences in the types of VCs among different tea samples (Figure 2b).

The functional groups of VCs in GABA sun-dried green tea and GABA raw Pu-erh tea could be divided into 13 categories, including 35 hydrocarbons, 26 terpenes, 22 esters, 12 alcohols, 12 heterocyclics, 11 ketones, 10 aromatics, 8 aldehydes, 4 phenols, 3 halogenated hydrocarbons, 3 acids, 1 nitrogenous compound, and 1 miscellaneous category. These 13 VCs were detected in SGT, PRT, CK, and CA samples, with a total of 145 VCs detected in 13 categories in SGT and PRT, 143 VCs detected in 13 categories in CK, and 148 VCs detected in 13 categories in CA. The 10 VCs with the highest contents in the PRT samples were caffeine (13.43 μg/g), linalool (9.11 μg/g), [2H3]-beta.-ionone (8.27 μg/g), L-alpha.-terpineol (6.97 μg/g), geraniol (5.50 μg/g), 2,3-dihydro-benzofuran (2.72 μg/g), anethole (6.37 μg/g), propane,2-chloro-2-nitro- (5.84 μg/g), 1-octen-3-ol (2.58 μg/g), and 1,3-benzenediol,5-pentyl- (1.88 μg/g). Compared with the SGT, the contents of the first six VCs increased significantly, and the others increased in the SGT. This study represents the first use of HS-SPME-GC-MS technology to detect and determine the VCs of GABA raw Pu-erh tea, demonstrating that the contents of terpenes such as linalool, [2H3]-beta.-ionone, L-alpha.-terpineol, and geraniol could be effectively increased by autoclave treatment after anaerobic treatment. At present, it is speculated that these four terpenes may be the key compounds contributing to the distinct aroma of GABA raw Pu-erh tea, which required further validation by OAV or ROAV.

According to the isoprene rules in organisms, the carbon units are condensed to form precursors for the synthesis of terpenes, which have basic functions in the growth and development of organisms and continue to form more terpenes through metabolic pathways [33]. According to existing research, terpenes are important contributors to the VCs of green tea, black tea, Pu-erh tea, and other teas, but they are rarely reported as the first-class compound category [34,35]. Therefore, the molecular configuration and metabolic pathway of terpenes can be used as an important direction for the study of characteristic aroma compounds in tea in the future. In this study, 26 terpenes were detected in SGT, PRT, CK, and CA samples. Compared with the SGT, the contents of 18 terpenes in the PRT increased significantly, and the total content of terpenes increased by 1.4 times, indicating that the autoclave technology contributed to the formation of terpenes in PRT. A variety of terpenes have been found and confirmed to be the characteristic aroma compounds in tea, such as linalool with a sweet, woody, flowery, and fruity aroma [36]; [2H3]-beta.-ionone with a woody and violet flowery aroma, accompanied by a fruity aroma [37]; L-alpha.-terpineol with a flowery and light grassy aroma [38]; and geraniol with a sweet and light rose aroma [39]. The aroma characteristics of these terpenes can help to better explain the aroma type of PRT. In addition, most of the increased terpenes have a relatively low OT [40], which can be used as a basis for further verification of whether these terpenes contribute to the formation of aroma in PRT.

OPLS-DA is generally used to distinguish the differences of variables between two groups. In this study, OPLS-DA was used to distinguish and analyze the different VCs between SGT and PRT samples (Figure 2c). A total of 68 differential VCs between the SGT and the PRT were screened (variable importance in projection (VIP) > 1, *p* < 0.05, fold change > 0). By comparing the SGT with the PRT (Figure 2d), 49 VCs were found to be up-regulated, including linalool, geraniol, alpha.-ionone, [2H3]-beta.-ionone, 6-methyl-6-(5-methylfuran-2-yl)heptan-2-one, 2-buten-1-one,1-(2,6,6-trimethyl-1,3-cyclohexadien-1-yl)-,(E)-, 1,3,8-p-menthatriene, undecane, 2,9-dimethyl-, hexadecanoic acid, methyl ester, alpha.-cadinol, and others; 19 VCs were down-regulated, including anethole, 1-octen-3-ol, 1-dodecanol, 3-methyl-tetradecane, hexadecane, 2-methyl-, phenylethyl alcohol, 2-methoxy-furan, cyclohexanol, 2,6-dimethyl-, (3R,6S)-2,2,6-trimethyl-6-vinyltetrahydro-2H-pyran-3-ol, diethyl phthalate, and others.

### 3.3. Key Active Compounds of GABA Sun-Dried Green Tea and GABA Raw Pu-Erh Tea Aroma

**Figure 3:**
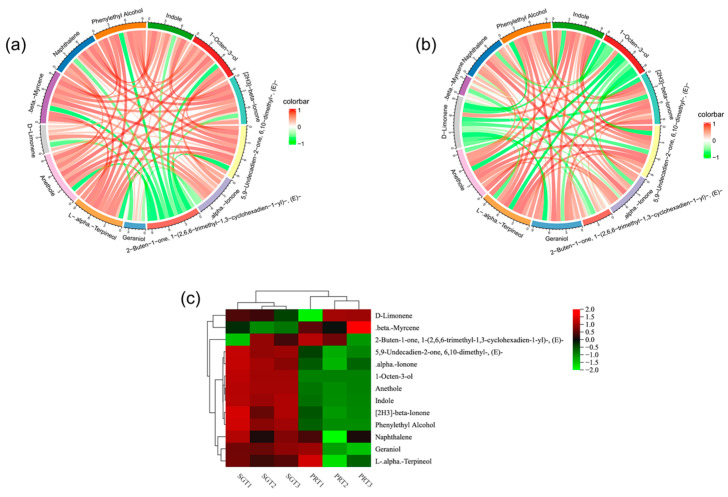
Based on the ROAV value, the correlation statistics of the characteristic aroma compounds of tea samples were carried out. SGT refers to GABA sun-dried green tea, and PRT refers to GABA raw Pu-erh tea. (**a**) Pearson correlation circle of characteristic aroma compounds of PRT. (**b**) Pearson correlation circle of characteristic aroma compounds of SGT. (**c**) Heat map of hierarchical clustering of relative odor activity value.

The types of VCs in SGT and PRT are very complex, and many of the VCs identified are odorless or have low odor activity with no contribution to or minimal effect on the overall aroma formation of tea samples. Therefore, the key active compounds of SGT and PRT aroma formation were screened to reveal the key role of VCs in the formation of tea aroma. A total of 14 VCs were screened based on ROAV and aroma types (Table 3). In the SGT, there were six VCs with ROAV > 1 and eight compounds with 0.1 < ROAV < 1. In the PRT, there were five VCs with ROAV > 1 and eight compounds with 0.1 < ROAV < 1. The Pearson correlation circle showed that there was a weak correlation between 2-buten-1-one,1-(2,6,6-trimethyl-1,3-cyclohexadien-1-yl)-, (E)- and other compounds in PRT, which was more prominent (Figure 3a). In SGT, 1-octen-3-ol and D-limonene showed a weak correlation with other compounds (Figure 3b). Further analysis of ROAV by construction of the cluster heat map showed that the key active compounds in SGT and PRT were significantly different (Figure 3c).

In the SGT, there were two types of VCs with ROAV > 90, which were linalool (woody, flowery, fruity (weak), and sweet (weak)) and 1-octen-3-ol (clean, fatty, and mushroomy). The VCs of 1 < ROAV < 90 included alpha.-ionone (flowery, sweet, and weak), 2-buten-1-one,1-(2,6,6-trimethyl-1,3-cyclohexadien-1-yl)-, (E)- (flowery, fruity, and lasting), geraniol (flowery, sweet, weak, and normal), and beta.-myrcene (fatty and weak). The compounds with 0.1 < ROAV < 1 included [2H3]-beta.-ionone (woody and flowery), 5,9-undecadien-2-one,6,10-dimethyl-, (E)- (fresh, flowery, and sweet (weak)), L-alpha.-terpineol (woody, flowery, and weak), anethole (licorice), D-limonene (flowery, lemony, and weak), naphthalene (aromatic and normal), phenylethyl alcohol (sweet (weak) and flowery (weak)), and indole (flowery and fresh). The aroma types of SGT mainly included flowery, clean, fatty, fruity (weak), and sweet (weak). Because there were two main VCs with ROAV > 90, and the two compounds show completely different aroma types, SGT forms the aroma characteristics of flowery and pure aroma.

In the PRT group, linalool (woody, flowery, fruity (weak), and sweet (weak)) was the only VC with ROAV > 90. The VCs of 1 < ROAV < 90 included 2-buten-1-one,1-(2,6,6-trimethyl-1,3-cyclohexadien-1-yl)-, (E)- (flowery, fruity, and lasting), geraniol (flowery, sweet, weak, and normal), beta.-myrcene (fatty (weak)), and 1-octen-3-ol (clean, fatty, and mushroomy). The compounds with 0.1 < ROAV < 1 included [2H3]-beta.-ionone (woody and flowery), 5,9-undecadien-2-one,6,10-dimethyl-, (E)- (fresh, flowery, and sweet (weak)), alpha.-ionone (flowery, sweet, and weak), L-alpha.-terpineol (woody, flowery, and weak), anethole (licorice), D-limonene (flowery, lemony, and weak), naphthalene (aromatic and normal), and phenylethyl alcohol (sweet (weak) and flowery (weak)). Compared with SGT, the ROAV of linalool and 1-octen-3-ol in PRT showed a fourfold difference, and the aroma of 1-octen-3-ol was relatively weakened so that the aroma of PRT showed pure characteristics. Because the main VCs in PRT have better aroma persistence, such as 2-buten-1-one,1-(2,6,6-trimethyl-1,3-cyclohexadien-1-yl)-,(E)-, PRT shows persistent aroma characteristics. These results indicate that autoclave technology can improve the purity and persistence of tea aroma and effectively improve the quality of tea aroma. From the perspective of aroma types, the main VCs in PRT were dominated by linalool, which relatively weakened the VCs exhibiting aroma characteristics such as clean, refreshing, and fatty aroma, and finally conferred PRT with a flowery and fruity aroma characterized as light and sweet.

Linalool (ROAV = 100) in SGT had the greatest contribution to the aroma, followed by 1-octen-3-ol (ROAV = 96.51), geraniol (ROAV = 13.54), 2-buten-1-one,1-(2,6,6-trimethyl-1,3-cyclohexadien-1-yl)-,(E)-(ROAV = 10.51), beta.-myrcene (ROAV = 1.16), and alpha.-ionone (ROAV = 1.09). According to previous screening results of GC-MS and differential VCs, linalool and alpha.-ionone greatly contributed to the aroma of sun-dried green tea, which was consistent with the results of this study [41]. However, this study also found other compounds that contribute more to the aroma, such as 2-buten-1-one,1-(2,6,6-trimethyl-1,3-cyclohexadien-1-yl)-,(E)- and beta.-myrcene; these compounds may be the reason why GABA sun-dried green tea has no obvious sun flavor. Linalool (ROAV = 100) had the greatest contribution to the aroma in PRT, followed by 1-octen-3-ol (ROAV = 21.21), geraniol (ROAV = 12.11), 2-buten-1-one,1-(2,6,6-trimethyl-1,3-cyclohexadien-1-yl)-,(E)- (ROAV = 10.68), and beta.-myrcene (ROAV = 1.26). In addition to linalool, methoxy compounds and ketones also contributed to the aroma of raw Pu-erh tea [42]. This study found that the compounds with the main contribution to the aroma of GABA raw Pu-erh tea were terpenes, which indicated that anaerobic technology could significantly change the flavor of raw Pu-erh tea. In previous studies on the aroma compounds of sun-dried green tea, most of the compounds were identified to contribute to the aroma, such as methoxy, aldehydes, and ketones [43]. However, terpenes such as 2-buten-1-one,1-(2,6,6-trimethyl-1,3-cyclohexadien-1-yl)-,(E)- and beta.-myrcene were identified to have a great contribution to the aroma of the tea in this study for the first time, with an ROAV of more than one in both PRT and SGT, although the ROAV of the two compounds in PRT was higher than that in SGT. This indicates that these compounds play an important role in aroma formation, which is not consistent with previous studies demonstrating that the terpenes linalool, geraniol, and alpha.-ionone are important contributors to the aroma formation of sun-dried green tea and raw Pu-erh tea [44]. This difference may be due to the fact that previous studies tested a lower content of GABA sun-dried green tea and GABA raw Pu-erh tea. Another reason may be that there are differences between tea varieties. In this study, the contribution of 2-buten-1-one,1-(2,6,6-trimethyl-1,3-cyclohexadien-1-yl)-,(E)- and beta.-myrcene to the aroma formation of GABA sun-dried green tea and GABA raw Pu-erh tea was clarified by ROAV; however, determining the effect of dynamic changes of VCs on the quality of the tea requires further study.

### 3.4. Potential Markers of Differential Aroma

Finding and identifying potential marker aroma compounds is an important quantitative research method in the study of flavor differences [45]. There were significant differences in aroma characteristics between SGT and PRT. OPLS-DA and ROAV analysis (VIP > 1, ROAV > 1, *p* < 0.05, fold change > 0) identified differential VCs of SGT and PRT, including linalool, 1-octen-3-ol, geraniol, 2-buten-1-one,1-(2,6,6-trimethyl-1,3-cyclohexadien-1-yl)-,(E)-, and beta.-myrcene. These compounds may therefore be used as potential markers of SGT and PRT aroma. Among them, linalool, geraniol, and 1-octen-3-ol were confirmed as potential markers for the aroma of sun-dried green tea and raw Pu-erh tea in a previous study [9].

The production of potential markers is usually related to the variety, processing technology, or unique active ingredients. The identification of potential markers of tea can help tea quality control or test repeatability [46]. According to previous studies, potential marker aroma compounds may be formed during tea storage or processing, and even biological pathways such as oxidation and degradation dominated by environmental factors may also promote the formation of potential marker aroma compounds [47,48]. According to the five potential marker aroma compounds found in this study, we believe that it is likely to be due to anaerobic treatment. Potential marker aroma compounds have practical significance for explaining the aroma results of this study and will provide a reference for future research on aroma compounds of GABA tea.

### 3.5. Metabolic Evolution Pathway of Main Aroma Compounds

**Figure 4:**
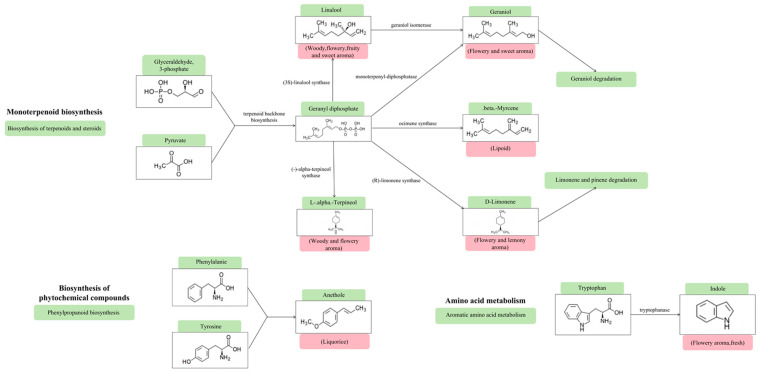
Metabolic evolution pathway of main flavor compounds in GABA sun-dried green tea and GABA raw Pu-erh tea.

The main VCs in SGT and PRT were terpenes and alcohols, which was similar to the results of previous studies [49]. KEGG pathway analysis was used to further explore the metabolic pathways for the formation of key aroma compounds in SGT and PRT. The results showed that the formation of major VCs mainly involves monoterpenoid biosynthesis, biosynthesis of phytochemical compounds, and amino acid metabolism (Figure 4).

**Figure 5:**
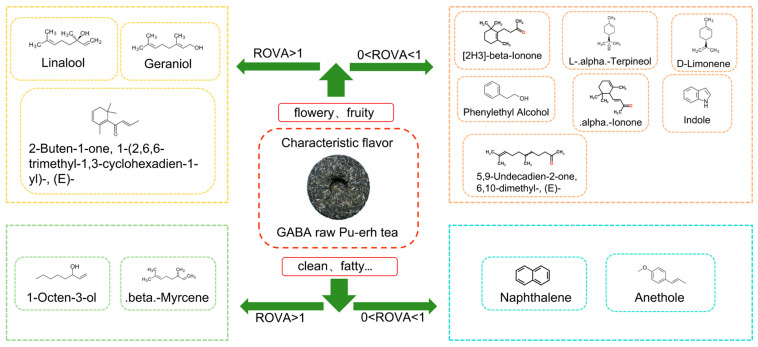
Characteristic aroma compounds and characteristic aroma in GABA raw Pu-erh tea.

Combined with the characteristic aroma compounds map and metabolic pathway map of PRT (Figure 5), it can be found that terpenes in SGT and PRT are mainly formed through monoterpenoid biosynthesis. Most of the terpenes in tea have woody, flowery, and fruity aromas, which can better coordinate the aroma of SGT and PRT. According to a previous study, the product of glycoside hydrolysis, geranyl diphosphate, is an important precursor of terpenoids [50]. Hydrolases hydrolyze glycoside to form geranyl diphosphate in the process of water loss and anaerobic digestion of SGT and PRT, which leads to the formation of various terpenes in the tea through the enzymatic action of corresponding synthases. In addition, linalool forms geraniol under the action of isomerase. Indole is mainly formed through the amino acid metabolism pathway, and anethole is mainly formed through the biosynthesis of phytochemical compounds, which has a certain contribution to SGT aroma; on the one hand, it enriches the SGT aroma type, and on the other hand, it affects the purity of the SGT aroma. However, the contribution of indole to the PRT aroma was not significant. A previous study found that high-temperature drying will lead to the degradation of a large number of amino acids in black tea [51]. In the process of SGT deactivation and autoclaving, tryptophan, as a precursor for the synthesis of indole, is greatly degraded, thus reducing the contribution of indole to the PRT aroma.

In addition, some VCs are produced by non-enzymatic reactions. For example, 2-buten-1-one,1-(2,6,6-trimethyl-1,3-cyclohexadien-1-yl)-,(E)- is mainly formed by the non-enzymatic reaction of neoxanthin under acidic conditions such as thermal degradation or oxidation [52]. Therefore, after autoclaving, the content of 2-buten-1-one,1-(2,6,6-trimethyl-1,3-cyclohexadien-1-yl)-,(E)- increased significantly, which was beneficial to the formation of the floral and fruity aroma of PRT. Linoleic acid is oxidized by lipoxygenase and then reduced by alcohol dehydrogenase to form 1-octen-3-ol; however, the key enzyme is still unclear [53]. A previous study showed that the content of 1-octen-3-ol in wheat food significantly reduced after steam heating or baking heating [54]. Although the specific degradation mechanism remains unclear, this may explain the difference in 1-octen-3-ol between SGT and PRT.

## 4. Conclusions

In summary, the main VCs in SGT and PRT were formed by monoterpenoid biosynthesis, biosynthesis of phytochemical compounds, and amino acid metabolism during processing. We believe that terpenes play an important role in promoting the aroma of SGT and PRT, mainly including linalool, geraniol, 2-buten-1-one,1-(2,6,6-trimethyl-1,3-cyclohexadien-1-yl)-,(E)-, and beta.-myrcene, resulting in flowery and fruity aroma characteristics. Compared with PRT, 1-octen-3-ol, indole, and anethole may contribute to the more impure aroma of SGT, which may be related to the autoclave processing technology. In addition, the qualitative and quantitative results of VCs were quite different between CK and CA samples, indicating that anaerobic technology could effectively change the aroma compounds and contents in fresh tea leaves. Overall, the findings of this study provide a basis for the formation of flowery and fruity aromas, help to clarify the characteristic aroma compounds, and serve as a guide to further improve the quality of SGT and PRT.

In the future, aroma recombination experiments can be explored, and response surface experiments can be designed to provide more clear evidence for determining the actual aroma contribution compounds and to determine improved quality optimization methods. Finally, the dynamic change mechanism of terpenoids and other compounds that have significant contributions to aroma in tea processing remains to be elucidated.

## Figures and Tables

**Table 1:** Sensory evaluation results of GABA sun-dried green tea and GABA raw Pu-erh tea.

Sample	Appearance	s	Aroma	s	Beverage Color	s	Taste	s	Infused Leaves	s	Total
SGT	Approach tight, yellowish green	86	Flowery aroma, fruity aroma (inferior), fragrant, high, pure, lasting	89	Lightly apricot, bright	90	Mellow, thick	86	Soft, bright	91	87.65
PRT	Normal cake, yellowish green	88	Flowery aroma, fruity aroma and sweet aroma, fragrant, high, more pure and lasting	90	Greenish yellow, bright	91	Sweet, fresh, thick	90	More soft and bright	90	89.7

**Table 2:** Volatile compound composition and absolute contents.

No.	Aroma Compounds	Retention Time	Retention Index	Identification Methods	Absolute Content (μg/g)			
					SGT	PRT	CK	CA
1	[2H3]-beta.-Ionone	16.66	1477.90	MS, RI	7.41 ± 0.18 ^b^	8.27 ± 0.14 ^a^	7.96 ± 0.48 ^a^	6.79 ± 0.15 ^c^
2	Phytol, acetate	22.32	1837.31	MS, RI	0.16 ± 0.00 ^b^	0.20 ± 0.01 ^a^	0.06 ± 0.01 ^c^	0.16 ± 0.00 ^c^
3	Phthalic acid, butyl hept-4-yl ester	23.09	1958.70	MS, RI	0.30 ± 0.00 ^c^	0.24 ± 0.02 ^c^	0.39 ± 0.09 ^b^	1.05 ± 0.01 ^a^
4	Linoleic acid ethyl ester	24.50	2160.32	MS, RI	0.12 ± 0.01 ^b^	0.18 ± 0.03 ^a^	——	0.03 ± 0.00 ^c^
5	Isophytol	23.08	1946.90	MS, RI	0.07 ± 0.00 ^b^	0.08 ± 0.01 ^a^	0.04 ± 0.01 ^c^	0.07 ± 0.00 ^ab^
6	Phytol	22.80	2113.79	MS, RI	0.12 ± 0.01 ^a^	0.15 ± 0.05 ^a^	0.04 ± 0.01 ^b^	0.11 ± 0.01 ^a^
7	3,7,11,15-Tetramethyl-2-hexadecen-1-ol	23.45	1879.20	MS, RI	0.09 ± 0.00 ^b^	0.11 ± 0.01 ^a^	0.03 ± 0.01 ^c^	0.08 ± 0.00 ^b^
8	9,12-Octadecadienoic acid (Z,Z)-, methyl ester	23.67	2092.49	MS, RI	0.15 ± 0.01 ^b^	0.20 ± 0.03 ^a^	——	0.01 ± 0.00 ^c^
9	Hexadecanoic acid, ethyl ester	24.40	1990.70	MS, RI	0.73 ± 0.02 ^b^	1.05 ± 0.08 ^a^	0.01 ± 0.00 ^d^	0.14 ± 0.00 ^c^
10	Hexadecane, 2,6,10,14-tetramethyl-	22.09	1805.46	MS, RI	0.15 ± 0.00 ^c^	0.02 ± 0.00 ^d^	0.16 ± 0.01 ^b^	0.20 ± 0.00 ^a^
11	Hexadecane, 2,6,11,15-tetramethyl-	15.29	1745.74	MS, RI	0.16 ± 0.00 ^c^	0.06 ± 0.00 ^d^	0.24 ± 0.02 ^b^	0.26 ± 0.01 ^a^
12	9,12,15-Octadecatrienoic Acid, (Z,Z,Z)-	24.56	2167.24	MS, RI	0.06 ± 0.01 ^b^	0.09 ± 0.02 ^a^	——	0.01 ± 0.00 ^c^
13	Sulfurous acid, 2-ethylhexyl hexyl ester	24.45	1998.17	MS, RI	0.01 ± 0.00 ^b^	0.02 ± 0.00 ^a^	0.02 ± 0.01 ^a^	0.02 ± 0.00 ^ab^
14	7,9-Di-tert-butyl-1-oxaspiro(4,5)deca-6,9-diene-2,8-dione	23.59	1907.29	MS, RI	0.05 ± 0.00 ^b^	0.05 ± 0.00 ^b^	0.07 ± 0.01 ^a^	0.05 ± 0.01 ^b^
15	Hexadecanoic acid, methyl ester	23.67	1923.63	MS, RI	1.23 ± 0.03 ^b^	1.62 ± 0.08 ^a^	0.12 ± 0.02 ^d^	0.30 ± 0.01 ^c^
16	Pentadecane, 2,6,10,14-tetramethyl-	22.33	1701.98	MS, RI	0.29 ± 0.02 ^c^	0.03 ± 0.00 ^d^	0.38 ± 0.04 ^b^	0.50 ± 0.05 ^a^
17	2-Pentadecanone, 6,10,14-trimethyl-	21.33	1842.46	MS, RI	0.38 ± 0.00 ^b^	0.56 ± 0.03 ^a^	0.17 ± 0.04 ^c^	0.11 ± 0.00 ^d^
18	(Z)-9,17-Octadecadienal	24.21	2098.35	MS, RI	0.07 ± 0.00 ^b^	0.09 ± 0.01 ^a^	——	0.01 ± 0.00 ^c^
19	Farnesol, acetate	18.28	1560.48	MS, RI	0.16 ± 0.00 ^b^	0.19 ± 0.00 ^a^	0.17 ± 0.02 ^ab^	0.17 ± 0.00 ^ab^
20	Heptadecane, 2-methyl-	20.42	1674.10	MS, RI	0.13 ± 0.01 ^b^	0.07 ± 0.01 ^c^	0.18 ± 0.05 ^a^	0.15 ± 0.01 ^ab^
21	Heptadecane, 3-methyl-	21.11	1772.65	MS, RI	0.15 ± 0.00 ^b^	0.04 ± 0.00 ^c^	0.27 ± 0.04 ^a^	0.26 ± 0.00 ^a^
22	Pentadecane, 2,6,10-trimethyl-	21.17	1798.71	MS, RI	0.13 ± 0.00 ^b^	0.15 ± 0.02 ^a^	0.18 ± 0.03 ^c^	0.17 ± 0.01 ^c^
23	2-Oxobicyclo(3.2.2)nona-3,6-dien-1-yl benzoate	20.48	1588.30	MS, RI	0.09 ± 0.00 ^b^	0.09 ± 0.01 ^b^	0.10 ± 0.01 ^b^	0.21 ± 0.01 ^a^
24	Hexadecane, 4-methyl-	19.85	1660.99	MS, RI	0.08 ± 0.01 ^c^	0.04 ± 0.00 ^d^	0.12 ± 0.01 ^b^	0.14 ± 0.00 ^a^
25	Hexadecane, 2-methyl-	19.89	1666.84	MS, RI	0.10 ± 0.00 ^c^	0.05 ± 0.01 ^d^	0.14 ± 0.01 ^b^	0.16 ± 0.01 ^a^
26	Heptadecane	20.65	1698.50	MS, RI	0.40 ± 0.02 ^b^	0.36 ± 0.03 ^b^	0.36 ± 0.05 ^b^	0.47 ± 0.02 ^a^
27	4-ethyl-Tetradecane	18.58	1542.70	MS, RI	0.05 ± 0.00 ^c^	0.05 ± 0.01 ^c^	0.10 ± 0.01 ^b^	0.13 ± 0.00 ^a^
28	Hexadecane	20.04	1598.46	MS, RI	0.83 ± 0.01 ^c^	0.53 ± 0.03 ^d^	0.98 ± 0.06 ^b^	1.17 ± 0.02 ^a^
29	Pentadecane, 4-methyl-	20.26	1556.14	MS, RI	0.02 ± 0.00 ^c^	0.02 ± 0.00 ^d^	0.03 ± 0.00 ^b^	0.04 ± 0.00 ^a^
30	Nonane, 2,2,4,4,6,8,8-heptamethyl-	17.33	1409.57	MS, RI	0.07 ± 0.00 ^c^	0.09 ± 0.01 ^c^	0.11 ± 0.02 ^b^	0.16 ± 0.01 ^a^
31	3-methyl-Pentadecane	18.99	1569.34	MS, RI	0.34 ± 0.01 ^c^	0.27 ± 0.01 ^d^	0.57 ± 0.04 ^b^	0.72 ± 0.01 ^a^
32	2,6,10-Trimethyltridecane	17.96	1459.04	MS, RI	0.85 ± 0.01 ^b^	1.56 ± 0.18 ^a^	0.57 ± 0.04 ^c^	0.37 ± 0.00 ^c^
33	diethyl(decyloxy)-Borane	9.55	999.10	MS, RI	0.01 ± 0.00 ^b^	0.01 ± 0.01 ^b^	0.01 ± 0.00 ^b^	0.03 ± 0.00 ^a^
34	Pentadecanal	12.93	1716.20	MS, RI	0.01 ± 0.00 ^a^	0.01 ± 0.00 ^a^	0.01 ± 0.00 ^a^	0.01 ± 0.00 ^a^
35	Nerolidol 1	13.43	1911.51	MS, RI	0.06 ± 0.00 ^b^	0.07 ± 0.01 ^a^	0.05 ± 0.01 ^b^	0.03 ± 0.00 ^c^
36	(3R,3aS,6S,7R)-3,6,8,8-Tetramethyloctahydro-1H-3a,7-methanoazulen-6-ol	19.10	1618.06	MS, RI	0.03 ± 0.00 ^b^	0.01 ± 0.00 ^c^	0.04 ± 0.00 ^a^	0.04 ± 0.00 ^a^
37	alpha.-Cadinol	19.88	1664.61	MS, RI	0.05 ± 0.00 ^b^	0.06 ± 0.00 ^a^	0.03 ± 0.00 ^c^	0.06 ± 0.00 ^a^
38	Diethyl Phthalate	12.87	1586.64	MS, RI	0.17 ± 0.00 ^c^	0.02 ± 0.00 ^d^	1.09 ± 0.15 ^b^	1.59 ± 0.02 ^a^
39	(3E,7E)-4,8,12-Trimethyltrideca-1,3,7,11-tetraene	19.58	1572.66	MS, RI	0.09 ± 0.00 ^c^	0.15 ± 0.01 ^a^	0.06 ± 0.01 ^d^	0.11 ± 0.00 ^b^
40	(1R,4S,9aS)-1-Methyl-4-((Z)-pent-2-en-4-yn-1-yl)octahydro-1H-quinolizine	19.75	1565.64	MS, RI	0.29 ± 0.01 ^c^	0.44 ± 0.01 ^a^	0.31 ± 0.03 ^c^	0.36 ± 0.01 ^b^
41	3-methyl-Tetradecane	19.71	1654.55	MS, RI	0.05 ± 0.00 ^c^	0.03 ± 0.00 ^d^	0.08 ± 0.00 ^b^	0.10 ± 0.00 ^a^
42	Tetradecane, 4-methyl-	17.56	1419.36	MS, RI	0.03 ± 0.00 ^b^	0.03 ± 0.00 ^a^	0.05 ± 0.01 ^c^	0.07 ± 0.00 ^c^
43	Pentadecane	18.65	1498.34	MS, RI	0.25 ± 0.00 ^b^	0.34 ± 0.02 ^a^	0.30 ± 0.02 ^a^	0.34 ± 0.02 ^a^
44	2,2’,5,5’-tetramethyl-1,1’-Biphenyl	19.78	1660.37	MS, RI	0.03 ± 0.00 ^c^	0.05 ± 0.00 ^b^	0.05 ± 0.00 ^b^	0.08 ± 0.00 ^a^
45	6-Methyl-6-(5-methylfuran-2-yl)heptan-2-one	17.45	1416.72	MS, RI	0.07 ± 0.00 ^b^	0.14 ± 0.00 ^a^	0.07 ± 0.00 ^b^	0.05 ± 0.00 ^c^
46	4’,6’-Dimethoxy-2’,3’-dimethylacetophenone	19.24	1628.78	MS, RI	0.08 ± 0.00 ^b^	0.12 ± 0.01 ^a^	0.05 ± 0.01 ^c^	0.01 ± 0.00 ^d^
47	2,4-Di-tert-butylphenol	18.79	1504.35	MS, RI	0.74 ± 0.16 ^b^	0.67 ± 0.15 ^b^	1.41 ± 0.21 ^a^	0.75 ± 0.15 ^b^
48	2-(2-butoxyethoxy)-Ethanol,acetate	12.45	1167.75	MS, RI	1.27 ± 0.03 ^b^	1.49 ± 0.03 ^a^	1.39 ± 0.11 ^ab^	1.30 ± 0.04 ^b^
49	3-Hexen-1-ol benzoate	19.09	1573.04	MS, RI	0.15 ± 0.00 ^c^	0.19 ± 0.01 ^b^	0.11 ± 0.01 ^d^	0.25 ± 0.00 ^a^
50	Hexanoic acid, hexyl ester	17.32	1382.70	MS, RI	0.04 ± 0.00 ^b^	0.04 ± 0.00 ^b^	0.02 ± 0.00 ^c^	0.27 ± 0.00 ^a^
51	alpha.-Calacorene	18.89	1545.24	MS, RI	0.09 ± 0.00 ^b^	0.13 ± 0.00 ^a^	0.07 ± 0.01 ^c^	0.10 ± 0.00 ^b^
52	Tetradecane	17.38	1398.41	MS, RI	0.40 ± 0.00 ^b^	0.44 ± 0.06 ^a^	0.59 ± 0.06 ^c^	0.81 ± 0.01 ^c^
53	Dodecane, 4,6-dimethyl-	16.76	1319.70	MS, RI	0.10 ± 0.00 ^c^	0.14 ± 0.03 ^bc^	0.16 ± 0.03 ^b^	0.23 ± 0.00 ^a^
54	Tridecane, 2-methyl-	18.43	1493.53	MS, RI	0.23 ± 0.00 ^b^	0.25 ± 0.02 ^b^	0.10 ± 0.01 ^c^	0.27 ± 0.00 ^a^
55	3,5-Dimethyldodecane	17.08	1368.99	MS, RI	0.13 ± 0.00 ^c^	0.20 ± 0.03 ^b^	0.23 ± 0.04 ^b^	0.36 ± 0.00 ^a^
56	Hexanoic acid, 3-hexenyl ester, (Z)-	17.30	1377.24	MS, RI	0.33 ± 0.00 ^c^	0.34 ± 0.02 ^c^	0.48 ± 0.05 ^b^	1.23 ± 0.02 ^a^
57	(E)-Hexanoic acid, 2-hexenyl ester	17.37	1385.40	MS, RI	0.11 ± 0.00 ^b^	0.12 ± 0.01 ^b^	0.05 ± 0.01 ^c^	0.28 ± 0.01 ^a^
58	5,9-Undecadien-2-one, 6,10-dimethyl-, (E)-	17.88	1445.93	MS, RI	1.38 ± 0.01 ^b^	1.66 ± 0.04 ^a^	1.13 ± 0.04 ^c^	0.79 ± 0.02 ^d^
59	1-Oxaspiro [4.5]dec-6-ene, 2,6,10,10-tetramethyl-	16.51	1302.26	MS, RI	0.17 ± 0.00 ^b^	0.26 ± 0.05 ^a^	0.16 ± 0.03 ^b^	0.24 ± 0.00 ^a^
60	Caffeine	23.19	1853.02	MS, RI	11.83 ± 0.50 ^b^	13.43 ± 0.62 ^ab^	15.52 ± 2.04 ^a^	12.74 ± 0.60 ^b^
61	alpha.-Ionone	17.46	1423.20	MS, RI	0.25 ± 0.00 ^b^	0.30 ± 0.01 ^a^	0.16 ± 0.00 ^c^	0.12 ± 0.01 ^d^
62	5-Methyl-2,4-diisopropylphenol	16.77	1337.69	MS, RI	0.09 ± 0.00 ^b^	0.16 ± 0.00 ^a^	0.08 ± 0.01 ^c^	0.04 ± 0.00 ^c^
63	2-(1,1-Dimethylethyl)-6-(1-methylethyl)phenol	15.47	1258.48	MS, RI	——	0.01 ± 0.00 ^a^	0.01 ± 0.00 ^b^	0.01 ± 0.00 ^b^
64	2-Buten-1-one, 1-(2,6,6-trimethyl-1,3-cyclohexadien-1-yl)-, (E)-	17.32	1380.04	MS, RI	0.06 ± 0.00 ^b^	0.08 ± 0.00 ^a^	0.09 ± 0.00 ^a^	0.05 ± 0.01 ^b^
65	4-(2,6,6-Trimethylcyclohexa-1,3-dienyl)but-3-en-2-one	17.65	1407.67	MS, RI	0.10 ± 0.00 ^d^	0.12 ± 0.00 ^c^	0.14 ± 0.02 ^b^	0.17 ± 0.01 ^a^
66	1-Dodecanol	19.94	1591.17	MS, RI	0.04 ± 0.00 ^c^	0.02 ± 0.00 ^d^	0.08 ± 0.01 ^b^	0.10 ± 0.00 ^a^
67	Undecane, 2,9-dimethyl-	17.01	1362.43	MS, RI	0.09 ± 0.01 ^c^	0.11 ± 0.01 ^b^	0.08 ± 0.01 ^c^	0.13 ± 0.00 ^a^
68	Nonane, 5-(2-methylpropyl)-	12.82	1162.73	MS, RI	0.03 ± 0.00 ^b^	0.03 ± 0.01 ^b^	0.03 ± 0.01 ^b^	0.06 ± 0.00 ^a^
69	Decane, 3-ethyl-3-methyl-	15.59	1260.38	MS, RI	0.08 ± 0.00 ^b^	0.09 ± 0.03 ^b^	0.10 ± 0.03 ^b^	0.18 ± 0.00 ^a^
70	Undecane, 4,6-dimethyl-	13.51	1211.40	MS, RI	0.08 ± 0.00 ^b^	0.11 ± 0.03 ^b^	0.10 ± 0.03 ^b^	0.18 ± 0.00 ^a^
71	Undecane, 5,7-dimethyl-	16.81	1329.38	MS, RI	0.03 ± 0.00 ^b^	0.04 ± 0.01 ^b^	0.05 ± 0.01 ^b^	0.07 ± 0.00 ^a^
72	Decane, 2,3,5-trimethyl-	16.53	1304.88	MS, RI	0.05 ± 0.00 ^c^	0.06 ± 0.01 ^c^	0.07 ± 0.01 ^b^	0.08 ± 0.00 ^a^
73	n-Valeric acid cis-3-hexenyl ester	13.76	1228.24	MS, RI	0.01 ± 0.00 ^c^	0.02 ± 0.00 ^bc^	0.02 ± 0.00 ^b^	0.06 ± 0.00 ^a^
74	2(4H)-Benzofuranone, 5,6,7,7a-tetrahydro-4,4,7a-trimethyl-, (R)-	19.02	1533.13	MS, RI	0.96 ± 0.03 ^b^	1.08 ± 0.04 ^a^	0.60 ± 0.07 ^c^	0.20 ± 0.00 ^d^
75	1,3-Benzenediol, 5-pentyl-	18.96	1518.37	MS, RI	1.88 ± 0.01 ^b^	1.88 ± 0.13 ^b^	3.04 ± 0.24 ^a^	2.04 ± 0.06 ^b^
76	Aspirin	22.67	1807.58	MS, RI	0.01 ± 0.00 ^a^	——	0.01 ± 0.00 ^b^	0.01 ± 0.00 ^a^
77	Butyl benzoate	19.87	1580.75	MS, RI	0.02 ± 0.00 ^bc^	0.02 ± 0.00 ^b^	0.02 ± 0.00 ^c^	0.04 ± 0.00 ^a^
78	1, 1, 5-Trimethyl-1, 2-dihydronaphthalene	16.99	1357.23	MS, RI	0.03 ± 0.00 ^c^	0.05 ± 0.00 ^b^	0.04 ± 0.00 ^c^	0.06 ± 0.00 ^a^
79	Ethyl 4-(ethyloxy)-2-oxobut-3-enoate	15.87	1284.27	MS, RI	0.03 ± 0.00 ^b^	0.03 ± 0.00 ^b^	0.03 ± 0.00 ^c^	0.04 ± 0.00 ^a^
80	Undecane, 5-methyl-	10.73	1053.58	MS, RI	0.07 ± 0.01 ^b^	0.09 ± 0.04 ^b^	0.08 ± 0.03 ^b^	0.16 ± 0.01 ^a^
81	Decane, 2,4-dimethyl-	16.09	1298.50	MS, RI	0.06 ± 0.00 ^c^	0.08 ± 0.02 ^bc^	0.10 ± 0.02 ^b^	0.13 ± 0.01 ^a^
82	trans-Linalool oxide (furanoid)	11.08	1070.91	MS, RI	0.97 ± 0.01 ^b^	1.29 ± 0.03 ^a^	1.17 ± 0.07 ^c^	1.36 ± 0.03 ^c^
83	(E)-2-Hexenoic acid, butyl ester	17.68	1437.17	MS, RI	——	——	——	0.01 ± 0.00 ^a^
84	(3R,6S)-2,2,6-Trimethyl-6-vinyltetrahydro-2H-pyran-3-ol	13.12	1175.18	MS, RI	0.98 ± 0.02 ^b^	0.80 ± 0.04 ^c^	0.43 ± 0.02 ^d^	1.22 ± 0.04 ^a^
85	2H-Pyran-2-one, tetrahydro-6-(2-pentenyl)-, (Z)-	18.30	1491.40	MS, RI	0.13 ± 0.01 ^a^	0.14 ± 0.02 ^a^	0.07 ± 0.01 ^b^	0.13 ± 0.01 ^a^
86	2,6-Octadienoic Acid, 3,7-dimethyl-, (E)-	16.86	1353.99	MS, RI	1.24 ± 0.01 ^a^	1.18 ± 0.30 ^a^	0.29 ± 0.05 ^b^	1.04 ± 0.06 ^a^
87	2,6,6-trimethyl-1-Cyclohexene-1-acetAldehyde	15.45	1257.39	MS, RI	0.04 ± 0.00 ^a^	0.03 ± 0.00 ^ab^	0.03 ± 0.00 ^b^	0.02 ± 0.00 ^c^
88	BenzAldehyde, 2,4-dihydroxy-3,6-dimethyl-	19.97	1593.22	MS, RI	0.02 ± 0.00 ^b^	0.02 ± 0.00 ^a^	0.01 ± 0.00 ^d^	0.01 ± 0.00 ^c^
89	2-Cyclopenten-1-one, 3-methyl-2-(2-pentenyl)-, (Z)-	17.44	1394.17	MS, RI	0.37 ± 0.01 ^a^	0.37 ± 0.04 ^a^	0.26 ± 0.01 ^c^	0.32 ± 0.01 ^b^
90	2-(formyloxy)-1-phenyl-Ethanone	19.65	1573.33	MS, RI	0.10 ± 0.00 ^c^	0.12 ± 0.00 ^b^	0.05 ± 0.00 ^d^	0.16 ± 0.00 ^a^
91	Nonanoic Acid	15.73	1268.14	MS, RI	0.52 ± 0.04 ^a^	0.42 ± 0.08 ^a^	0.41 ± 0.08 ^a^	0.27 ± 0.01 ^b^
92	Decane, 5-methyl-	9.86	1011.55	MS, RI	0.02 ± 0.00 ^b^	0.02 ± 0.00 ^a^	0.02 ± 0.01 ^c^	0.04 ± 0.00 ^c^
93	Decanal	13.46	1205.23	MS, RI	0.03 ± 0.00 ^a^	0.03 ± 0.00 ^a^	0.01 ± 0.00 ^b^	0.01 ± 0.00 ^b^
94	Geraniol	15.37	1249.83	MS, RI	4.11 ± 0.09 ^b^	5.50 ± 0.46 ^a^	5.27 ± 0.30 ^a^	5.76 ± 0.11 ^a^
95	L-alpha.-Terpineol	13.34	1197.22	MS, RI	4.79 ± 0.08 ^c^	6.97 ± 0.17 ^a^	6.40 ± 0.31 ^b^	6.52 ± 0.15 ^b^
96	2,6-Octadien-1-ol, 3,7-dimethyl-, (Z)-	13.70	1225.24	MS, RI	0.85 ± 0.03 ^b^	1.25 ± 0.08 ^a^	1.18 ± 0.08 ^a^	1.26 ± 0.02 ^a^
97	3-Cyclohexen-1-ol, 4-methyl-1-(1-methylethyl)-, (R)-	13.06	1182.77	MS, RI	0.20 ± 0.00 ^d^	0.25 ± 0.00 ^c^	0.30 ± 0.03 ^b^	0.35 ± 0.01 ^a^
98	Linalool	11.60	1101.31	MS, RI	6.06 ± 0.14 ^c^	9.11 ± 0.35 ^a^	7.61 ± 0.80 ^b^	9.10 ± 0.07 ^a^
99	1,5,7-Octatrien-3-ol, 3,7-dimethyl-	11.78	1103.50	MS, RI	1.13 ± 0.02 ^c^	1.71 ± 0.07 ^a^	1.36 ± 0.11 ^b^	1.38 ± 0.03 ^b^
100	2,6,6-trimethyl-1-Cyclohexene-1-carboxaldehyde	13.66	1220.95	MS, RI	0.35 ± 0.00 ^a^	0.37 ± 0.02 ^a^	0.21 ± 0.02 ^b^	0.12 ± 0.00 ^c^
101	2,6-Octadienal, 3,7-dimethyl-, (E)-	15.69	1267.57	MS, RI	0.14 ± 0.00 ^a^	0.12 ± 0.01 ^ab^	0.12 ± 0.01 ^ab^	0.11 ± 0.00 ^b^
102	Methyl salicylate	13.26	1194.38	MS, RI	0.57 ± 0.00 ^b^	0.53 ± 0.04 ^a^	0.56 ± 0.05 ^c^	0.87 ± 0.02 ^c^
103	Methyl anthranilate	16.75	1345.34	MS, RI	0.09 ± 0.00 ^b^	0.10 ± 0.02 ^b^	0.06 ± 0.00 ^c^	0.13 ± 0.00 ^a^
104	Ethanone, 1-(2-hydroxy-5-methylphenyl)-	16.50	1313.12	MS, RI	0.51 ± 0.01 ^b^	0.56 ± 0.06 ^b^	0.13 ± 0.01 ^c^	0.63 ± 0.02 ^a^
105	2-Methyl-7-exo-vinylbicyclo [4.2.0]oct-1(2)-ene	12.33	1144.57	MS, RI	0.02 ± 0.00 ^b^	0.03 ± 0.00 ^a^	0.03 ± 0.00 ^a^	0.03 ± 0.00 ^a^
106	Benzene, pentamethyl-	15.83	1280.53	MS, RI	0.05 ± 0.00 ^c^	0.07 ± 0.01 ^b^	0.07 ± 0.01 ^b^	0.14 ± 0.01 ^a^
107	Anethole	15.90	1287.43	MS, RI	11.39 ± 0.11 ^b^	6.37 ± 0.34 ^d^	14.32 ± 0.35 ^a^	7.89 ± 0.14 ^c^
108	4-Formyl-3,5-dimethyl-1H-pyrrole-2-carbonitrile	15.81	1277.95	MS, RI	0.06 ± 0.00 ^b^	0.09 ± 0.02 ^ab^	0.11 ± 0.02 ^a^	0.07 ± 0.01 ^b^
109	4H-Pyran-4-one, 2,3-dihydro-3,5-dihydroxy-6-methyl-	12.25	1148.70	MS, RI	——	0.01 ± 0.00 ^b^	0.01 ± 0.00 ^ab^	0.01 ± 0.00 ^a^
110	Naphthalene, 2-methyl-	16.33	1300.17	MS, RI	0.16 ± 0.00 ^d^	0.20 ± 0.00 ^c^	0.21 ± 0.01 ^b^	0.30 ± 0.01 ^a^
111	(+)-4-Carene	10.03	1016.79	MS, RI	0.09 ± 0.00 ^c^	0.14 ± 0.02 ^b^	0.13 ± 0.02 ^b^	0.22 ± 0.00 ^a^
112	D-Limonene	10.20	1029.13	MS, RI	0.73 ± 0.02 ^d^	1.09 ± 0.15 ^b^	0.93 ± 0.17 ^c^	1.52 ± 0.01 ^a^
113	beta.-Myrcene	9.38	989.67	MS, RI	0.78 ± 0.01 ^c^	1.27 ± 0.09 ^a^	1.01 ± 0.13 ^b^	1.39 ± 0.04 ^a^
114	(S)-(+)-alpha-Phellandrene	9.77	1006.64	MS, RI	0.09 ± 0.00 ^c^	0.14 ± 0.01 ^b^	0.13 ± 0.02 ^b^	0.19 ± 0.00 ^a^
115	2,4,6-Octatriene, 3,4-dimethyl-	11.84	1127.36	MS, RI	0.04 ± 0.00 ^d^	0.07 ± 0.01 ^b^	0.06 ± 0.01 ^c^	0.09 ± 0.00 ^a^
116	.gamma.-Terpinene	10.65	1058.03	MS, RI	0.15 ± 0.00 ^b^	0.18 ± 0.04 ^b^	0.17 ± 0.04 ^b^	0.34 ± 0.00 ^a^
117	beta.-Ocimene	10.49	1045.17	MS, RI	0.42 ± 0.02 ^b^	0.53 ± 0.06 ^a^	0.32 ± 0.04 ^c^	0.49 ± 0.01 ^ab^
118	Benzene, 1,2,3,5-tetramethyl-	12.07	1120.46	MS, RI	0.13 ± 0.00 ^c^	0.21 ± 0.05 ^b^	0.17 ± 0.04 ^bc^	0.32 ± 0.01 ^a^
119	1,3,8-p-Menthatriene	12.97	1130.49	MS, RI	0.02 ± 0.00 ^c^	0.04 ± 0.00 ^a^	0.03 ± 0.00 ^c^	0.03 ± 0.00 ^b^
120	Benzene, 1-methyl-3-(1-methylethyl)-	10.16	1024.38	MS, RI	0.14 ± 0.00 ^c^	0.21 ± 0.04 ^b^	0.19 ± 0.05 ^bc^	0.41 ± 0.01 ^a^
121	Benzene, 1-methyl-3-(1-methylethenyl)-	11.28	1091.00	MS, RI	0.08 ± 0.00 ^c^	0.13 ± 0.01 ^a^	0.10 ± 0.01 ^b^	0.14 ± 0.00 ^a^
122	Benzene, 4-ethenyl-1,2-dimethyl-	12.66	1149.59	MS, RI	0.05 ± 0.00 ^bc^	0.08 ± 0.01 ^b^	0.07 ± 0.01 ^b^	0.12 ± 0.00 ^a^
123	Cyclohexanol, 2,6-dimethyl-	11.99	1111.77	MS, RI	0.21 ± 0.00 ^a^	0.19 ± 0.01 ^b^	0.15 ± 0.01 ^c^	0.10 ± 0.00 ^d^
124	1-Octen-3-ol	9.11	980.83	MS, RI	7.80 ± 0.14 ^a^	2.58 ± 0.16 ^b^	1.51 ± 0.24 ^c^	0.28 ± 0.02 ^d^
125	Naphthalene	13.43	1188.68	MS, RI	1.06 ± 0.01 ^c^	1.49 ± 0.08 ^b^	1.46 ± 0.14 ^b^	2.10 ± 0.06 ^a^
126	3,5,5-trimethyl-2-Hexene	8.89	975.03	MS, RI	2.74 ± 0.02 ^a^	0.60 ± 0.05 ^b^	0.56 ± 0.07 ^b^	0.10 ± 0.01 ^c^
127	Benzyl chloride	9.90	1012.98	MS, RI	0.06 ± 0.00 ^b^	0.09 ± 0.01 ^a^	0.07 ± 0.01 ^b^	0.06 ± 0.00 ^b^
128	3-Formyl-4,5-dimethyl-pyrrole	10.52	1047.82	MS, RI	0.16 ± 0.00 ^c^	0.05 ± 0.00 ^d^	0.27 ± 0.03 ^b^	0.60 ± 0.02 ^a^
129	Propane, 2-chloro-2-nitro-	6.03	709.00	MS, RI	6.38 ± 0.20 ^a^	5.84 ± 0.49 ^a^	5.28 ± 0.53 ^a^	5.55 ± 0.91 ^a^
130	1,3-Cyclopentadiene, 5,5-dimethyl-1-ethyl-	7.54	841.47	MS, RI	0.04 ± 0.00 ^b^	0.07 ± 0.02 ^a^	0.03 ± 0.01 ^c^	0.03 ± 0.00 ^c^
131	Phenylethyl Alcohol	11.80	1114.93	MS, RI	0.43 ± 0.02 ^b^	0.29 ± 0.03 ^c^	0.20 ± 0.02 ^d^	0.63 ± 0.00 ^a^
132	Phenol, 3,5-dimethyl-	8.40	934.00	MS, RI	0.02 ± 0.00 ^b^	0.03 ± 0.00 ^a^	0.02 ± 0.00 ^c^	0.01 ± 0.00 ^c^
133	2,3-dihydro-Benzofuran	13.64	1219.63	MS, RI	1.91 ± 0.03 ^b^	2.72 ± 0.27 ^a^	1.20 ± 0.17 ^d^	1.57 ± 0.06 ^c^
134	Indole	16.01	1295.10	MS, RI	2.28 ± 0.03 ^a^	1.11 ± 0.11 ^c^	1.46 ± 0.07 ^b^	1.42 ± 0.04 ^b^
135	2-Heptanol	8.22	902.75	MS, RI	0.02 ± 0.00 ^a^	0.02 ± 0.01 ^a^	0.02 ± 0.00 ^b^	0.03 ± 0.00 ^a^
136	Heptanal	8.20	901.74	MS, RI	0.08 ± 0.01 ^a^	0.06 ± 0.01 ^b^	0.03 ± 0.00 ^c^	0.03 ± 0.01 ^c^
137	Cyclobutanone, 2,2,3-trimethyl-	6.44	751.40	MS, RI	0.02 ± 0.00 ^b^	0.03 ± 0.00 ^b^	0.04 ± 0.02 ^b^	0.11 ± 0.01 ^a^
138	5-methyl-1,2,5,6-Tetrahydropyridin-2-one	10.06	1020.73	MS, RI	0.19 ± 0.02 ^a^	0.11 ± 0.02 ^c^	0.16 ± 0.04 ^ab^	0.11 ± 0.02 ^bc^
139	Benzyl Alcohol	10.24	1036.04	MS, RI	0.36 ± 0.00 ^b^	0.44 ± 0.03 ^a^	0.33 ± 0.03 ^b^	0.43 ± 0.00 ^a^
140	BenzAldehyde	8.86	963.89	MS, RI	0.31 ± 0.01 ^a^	0.33 ± 0.03 ^a^	0.23 ± 0.02 ^b^	0.23 ± 0.01 ^b^
141	Dihydro-3-(2H)-thiophenone	8.67	955.93	MS, RI	0.02 ± 0.00 ^b^	0.02 ± 0.00 ^a^	0.01 ± 0.00 ^c^	0.02 ± 0.00 ^c^
142	3-Hexen-1-ol, (E)-	8.70	856.29	MS, RI	0.14 ± 0.02 ^c^	0.16 ± 0.01 ^b^	0.09 ± 0.01 ^d^	0.39 ± 0.01 ^a^
143	Hexanal	6.98	797.72	MS, RI	0.07 ± 0.00 ^ab^	0.08 ± 0.03 ^a^	0.05 ± 0.01 ^b^	0.04 ± 0.01 ^b^
144	3-Hexen-2-one	6.66	797.57	MS, RI	0.15 ± 0.02 ^a^	0.08 ± 0.07 ^ab^	0.07 ± 0.01 ^b^	0.13 ± 0.01 ^ab^
145	2-methoxy-Furan	6.64	797.09	MS, RI	0.12 ± 0.00 ^c^	0.08 ± 0.01 ^d^	0.19 ± 0.03 ^b^	0.27 ± 0.01 ^a^
146	Dimethylphosphinic fluoride	6.01	701.52	MS, RI	0.03 ± 0.00 ^a^	0.02 ± 0.00 ^ab^	0.02 ± 0.00 ^c^	0.02 ± 0.00 ^bc^
147	3-ethyl-1H-Pyrrole	7.11	809.67	MS, RI	0.05 ± 0.00 ^b^	0.02 ± 0.00 ^b^	0.16 ± 0.04 ^a^	0.19 ± 0.01 ^a^
148	1-Pentanol	6.56	769.54	MS, RI	0.51 ± 0.02 ^a^	——	0.28 ± 0.03 ^c^	0.34 ± 0.02 ^b^

Represents that the post-test has been carried out, different lowercase letters in the same line indicate significant difference (*p* < 0.05), and ‘—’ indicates that it is not detected. Each tea sample was measured in parallel 3 times, and all data were expressed as mean value ± SD.

**Table 3:** ROAV and aroma types of key aroma compounds.

No.	Index	ROAV		VIP	Fold Change	Aroma Type
		SGT	PRT			
1	[2H3]-beta.-Ionone	0.39 ± 0.02	0.29 ± 0.02	1.13	1.12	Woody, flowery
2	5,9-Undecadien-2-one, 6,10-dimethyl-, (E)-	0.68 ± 0.01	0.55 ± 0.03	1.19	1.20	Fresh, flowery, sweet (weak)
3	alpha.-Ionone	1.09 ± 0.02	0.88 ± 0.05	1.16	1.21	Flowery, sweet, weak
4	2-Buten-1-one, 1-(2,6,6-trimethyl-1,3-cyclohexadien-1-yl)-, (E)-	10.51 ± 0.69	10.68 ± 0.66	1.18	1.52	Flowery, fruity, lasting
5	Geraniol	13.54 ± 0.15	12.11 ± 1.50	1.13	1.34	Flowery, sweet, weak, and normal
6	L-alpha.-Terpineol	0.39 ± 0.00	0.38 ± 0.02	1.21	1.46	Woody, flowery, weak
7	Linalool	100.00 ± 0.00	100.00 ± 0.00	1.20	1.50	Woody, flowery, fruity (weak), sweet (weak)
8	Anethole	0.28 ± 0.01	0.11 ± 0.01	1.21	0.56	Licorice
9	D-Limonene	0.53 ± 0.01	0.53 ± 0.05	1.09	1.50	Flowery, lemony, weak
10	beta.-Myrcene	1.16 ± 0.03	1.26 ± 0.07	1.19	1.63	Fatty (weak)
11	Naphthalene	0.52 ± 0.01	0.49 ± 0.04	1.18	1.41	Aromatic, normal
12	Phenylethyl Alcohol	0.24 ± 0.02	0.11 ± 0.02	1.16	0.67	Sweet (weak), flowery (weak)
13	Indole	0.11 ± 0.00	0.04 ± 0.00	1.21	0.49	Flowery, fresh
14	1-Octen-3-ol	96.51 ± 4.17	21.21 ± 0.57	1.05	0.33	Clean, fatty, mushroomy

Each tea sample was measured in parallel for 3 times, and all data were expressed as mean value ± SD.

## Data Availability

Data is contained within the article and Supplementary Materials.

## Supplementary Materials

The following supporting information can be downloaded at https://www.mdpi.com/article/10.3390/foods12244512/s1, Figure S1: Heat map of volatile compounds.

## References

[1] Lewis R. (2012). Tea: History, Terroirs, Varieties.

[2] Wu X., Liu Y., Guo J., Wang J., Li M., Tan Y., Zheng Q., Feng Y. (2021). Differentiating Pu-erh raw tea from different geographical origins by 1 H-NMR and U-HPLC/Q-TOF-MS combined with chemometrics. J. Food Sci..

[3] Lv H., Zhang Y., Lin Z., Liang Y. (2013). Processing and chemical constituents of Pu-erh tea: A review. Food Res. Int..

[4] Ahmed S., Stepp J. (2013). Pu-erh Tea: Botany, Production, and Chemistry. Tea in Health and Disease Prevention.

[5] Zheng X., Li Q., Xiang L., Liang Y. (2016). Recent Advances in Volatiles of Teas. Molecules.

[6] Yang X., Liu Y., Mu L., Wang W., Zhan Q., Luo M., Tian H., Lv C., Li J. (2018). Discriminant research for identifying aromas of non-fermented Pu-erh tea from different storage years using an electronic nose. J. Food Process. Preserv..

[7] Xu S., Zeng X., Wu H., Shen S., Yang X., Deng W., Ning J. (2021). Characterizing volatile metabolites in raw Pu’er tea stored in wet-hot or dry-cold environments by performing metabolomic analysis and using the molecular sensory science approach. Food Chem..

[8] Fan X., Chen N., Cai F., Ren F., Zhong J., Wang D., Shi L., Ren D., Yi L. (2020). Effects of manufacturing on the volatile composition of raw Pu-erh tea with a focus on de-enzyming and autoclaving–compressing treatments. LWT.

[9] Feng T., Sun J., Wang K., Song S., Chen D., Zhuang H., Lu J., Li D., Meng X., Shi M. (2022). Variation in Volatile Compounds of Raw Pu-erh Tea upon Steeping Process by Gas Chromatography-Ion Mobility Spectrometry and Characterization of the Aroma-Active Compounds in Tea Infusion Using Gas Chromatography-Olfactometry-Mass Spectrometry. J. Agric. Food Chem..

[10] Tsushida T., Murai T. (1987). Conversion of Glutamic Acid to γ-Aminobutyric Acid in Tea Leaves under Anaerobic Conditions. Agric. Biol. Chem..

[11] Norio N., Katsuhiro H., Toshihiro M., Hirokazu F. (1988). An Improvement of the Qualities of Anaerobically Treated Tea (Gabaron Tea) by Heating. Chagyo Kenkyu Hokoku Tea Res. J..

[12] Ren T., Zheng P., Zhang K., Liao J., Xiong F., Shen Q., Ma Y., Fang W., Zhu X. (2021). Effects of GABA on the polyphenol accumulation and antioxidant activities in tea plants (*Camellia sinensis* L.) under heat-stress conditions. Plant Physiol. Biochem..

[13] Zhen Z. (2012). Study on flavour volatiles of γ-aminobutyric acid (GABA) green tea. Afr. J. Biotechnol..

[14] Li Y., Wu T., Deng X., Tian D., Ma C., Wang X., Li Y., Zhou H. (2023). Characteristic aroma compounds in naturally withered and combined withered γ-aminobutyric acid white tea revealed by HS-SPME-GC-MS and relative odor activity value. LWT.

[15] Yang S. (2012). Study on GABA *Pu’er* Tea. Master’s Thesis.

[16] Yang P., Yu M., Song H., Xu Y., Lin Y., Granvogl M. (2022). Characterization of Key Aroma-Active Compounds in Rough and Moderate Fire Wuyi Rock Tea by Sensory-Directed Flavor Analysis and Elucidation of the Influences of Roasting on Aroma. J. Agric. Food Chem..

[17] Zhu J., Chen F., Wang L., Niu Y., Xiao Z. (2017). Evaluation of the synergism among volatile compounds in Oolong tea infusion by odour threshold with sensory analysis and E-nose. Food Chem..

[18] Guo X., Ho C.T., Schwab W., Wan X. (2021). Aroma profiles of green tea made with fresh tea leaves plucked in summer. Food Chem..

[19] Fang Z.Y., Li G.Z., Gu Y., Wen C., Ye H., Ma J.L., Liang Z.Y., Yang L., Wu J.W., Chen H.Y. (2022). Flavour analysis of different varieties of camellia seed oil and the effect of the refining process on flavour substances. LWT.

[20] Xie J., Wang L., Deng Y., Yuan H., Zhu J., Jiang Y., Yang Y. (2023). Characterization of the key odorants in floral aroma green tea based on GC-E-Nose, GC-IMS, GC-MS and aroma recombination and investigation of the dynamic changes and aroma formation during processing. Food Chem..

[21] Wang Y., Li C., Zhao Y., Li L., Yang X., Wu Y., Chen S., Cen J., Yang S., Yang D. (2020). Novel insight into the formation mechanism of volatile flavor in Chinese fish sauce (Yu-lu) based on molecular sensory and metagenomics analyses. Food Chem..

[22] Hong X., Wang C., Jiang R., Hu T., Zheng X., Huang J., Liu Z., Li Q. (2022). Characterization of the Key Aroma Compounds in Different Aroma Types of Chinese Yellow Tea. Foods.

[23] (2018). Methodology for Sensory Evaluation of Tea.

[24] Li J., Wang J., Yao Y., Hua J., Zhou Q., Jiang Y., Deng Y., Yang Y., Wang J., Yuan H. (2020). Phytochemical comparison of different tea (*Camellia sinensis*) cultivars and its association with sensory quality of finished tea. LWT.

[25] Deng X., Huang G., Tu Q., Zhou H., Li Y., Shi H., Wu X., Ren H., Huang K., He X. (2021). Evolution analysis of flavor-active compounds during artificial fermentation of Pu-erh tea. Food Chem..

[26] Bi J., Li Y., Yang Z., Lin Z., Chen F., Liu S., Li C. (2022). Effect of different cooking times on the fat flavor compounds of pork belly. J. Food Biochem..

[27] Jin Q., Wang Z., Chen Y., Luo Y., Tian N., Liu Z., Huang J., Liu S. (2022). Transcriptomics analysis reveals the signal transduction mechanism of brassinolides in tea leaves and its regulation on the growth and development of Camellia sinensis. BMC Genom..

[28] Ou Y., Zhang Y., Qin L., Miao Y., Xiao L. (2019). Research progress on evaluation methods of tea color, aroma and quality. Sci. Technol. Food Ind..

[29] Qin Z., Pang X., Chen D., Cheng H., Hu X., Wu J. (2013). Evaluation of Chinese tea by the electronic nose and gas chromatography–mass spectrometry: Correlation with sensory properties and classification according to grade level. Food Res. Int..

[30] Li Y., Zhang J., Jia H., Pan Y., Xu Y., Wang Y., Deng W. (2023). Metabolite analysis and sensory evaluation reveal the effect of roasting on the characteristic flavor of large-leaf yellow tea. Food Chem..

[31] Su D., Xu T., Li Y., Zhou H. (2022). Flavor evolution in raw Pu-erh tea during manufacturing using different processing types. LWT.

[32] Biasetton N., Disegna M., Barzizza E., Salmaso L. (2023). A new adaptive membership function with CUB uncertainty with application to cluster analysis of Likert-type data. Expert Syst. Appl..

[33] Tholl D. (2015). Biosynthesis and biological functions of terpenoids in plants. Adv. Biochem. Eng. Biotechnol..

[34] Wen S., Sun L., Zhang S., Chen Z., Chen R., Li Z., Lai X., Zhang Z., Cao J., Li Q. (2023). The formation mechanism of aroma quality of green and yellow teas based on GC-MS/MS metabolomics. Food Res. Int..

[35] Lv S., Wu Y., Li C., Xu Y., Liu L., Meng Q. (2014). Comparative analysis of Pu-erh and Fuzhuan teas by fully automatic headspace solid-phase microextraction coupled with gas chromatography-mass spectrometry and chemometric methods. J. Agric. Food Chem..

[36] Matsubara E., Morikawa T., Kusumoto N., Hashida K., Matsui N., Ohira T. (2021). Subjective Effects of Inhaling Kuromoji Tea Aroma. Molecules.

[37] Xu J., Zhang Y., Yan F., Tang Y., Yu B., Chen B., Lu L., Yuan L., Wu Z., Chen H. (2022). Monitoring Changes in the Volatile Compounds of Tea Made from Summer Tea Leaves by GC-IMS and HS-SPME-GC-MS. Foods.

[38] Shao C., Zhang Y., Lv H., Zhang Z., Zeng J., Peng Q., Zhu Y., Lin Z. (2022). Aromatic profiles and enantiomeric distributions of chiral odorants in baked green teas with different picking tenderness. Food Chem..

[39] Wu H., Chen Y., Feng W., Shen S., Wei Y., Jia H., Wang Y., Deng W., Ning J. (2022). Effects of Three Different Withering Treatments on the Aroma of White Tea. Foods.

[40] Abbas F., Ke Y., Yu R., Yue Y., Amanullah S., Jahangir M.M., Fan Y. (2017). Volatile terpenoids: Multiple functions, biosynthesis, modulation and manipulation by genetic engineering. Planta.

[41] Zhang W., Liu C., Yang R., Zheng T., Zhao M., Ma L., Yan L. (2019). Comparison of volatile profiles and bioactive components of sun-dried Pu-erh tea leaves from ancient tea plants on Bulang Mountain measured by GC-MS and HPLC. J. Zhejiang Univ. Sci. B.

[42] Pang X., Yu W., Cao C., Yuan X., Qiu J., Kong F., Wu J. (2019). Comparison of Potent Odorants in Raw and Ripened Pu-erh Tea Infusions Based on Odor Activity Value Calculation and Multivariate Analysis: Understanding the Role of Pile Fermentation. J. Agric. Food Chem..

[43] Du L., Li J., Li W., Li Y., Li T., Xiao D. (2014). Characterization of volatile compounds of Pu-erh tea using solid-phase microextraction and simultaneous distillation–extraction coupled with gas chromatography–mass spectrometry. Food Res. Int..

[44] Shen S., Wu H., Li T., Sun H., Wang Y., Ning J. (2023). Formation of aroma characteristics driven by volatile components during long-term storage of An tea. Food Chem..

[45] Toci A., Farah A. (2014). Volatile fingerprint of Brazilian defective coffee seeds: Corroboration of potential marker compounds and identification of new low quality indicators. Food Chem..

[46] Hu C., Li D., Ma Y., Zhang W., Lin C., Zheng X., Liang Y., Lu J. (2018). Formation mechanism of the oolong tea characteristic aroma during bruising and withering treatment. Food Chem..

[47] Guo X., Ho C., Schwab W., Song C., Wan X. (2019). Aroma compositions of large-leaf yellow tea and potential effect of theanine on volatile formation in tea. Food Chem..

[48] Su D., He J., Zhou Y., Li Y., Zhou H. (2022). Aroma effects of key volatile compounds in Keemun black tea at different grades: HS-SPME-GC-MS, sensory evaluation, and chemometrics. Food Chem..

[49] Liu M., Hu C., Lu Y., Zhang H., Zhang Z. (2013). Research progress on aroma components of *Pu’er* tea. Chin. Tea Process..

[50] Wang D., Yoshimura T., Kubota K., Kobayashi A. (2000). Analysis of glycosidically bound aroma precursors in tea leaves. 1. Qualitative and quantitative analyses of glycosides with aglycons as aroma compounds. J. Agric. Food Chem..

[51] Zhou J., Fang T., Li W., Jiang Z., Zhou T., Zhang L., Yu Y. (2022). Widely targeted metabolomics using UPLC-QTRAP-MS/MS reveals chemical changes during the processing of black tea from the cultivar *Camellia sinensis* (L.) O. Kuntze cv. Huangjinya. Food Res. Int..

[52] Ho C., Zheng X., Li S. (2015). Tea aroma formation. Food Sci. Hum. Wellness.

[53] Wang K., Liu F., Liu Z., Huang J., Xu Z., Li Y., Yang X. (2011). Comparison of catechins and volatile compounds among different types of tea using high performance liquid chromatograph and gas chromatograph mass spectrometer. Int. J. Food Sci. Technol..

[54] Klensporf D., Jeleń H.H. (2008). Effect of heat treatment on the flavor of oat flakes. J. Cereal Sci..

