# Supplementary material for: The Characteristic Aroma Compounds of GABA Sun-Dried Green Tea and Raw Pu-Erh Tea Determined by Headspace Solid-Phase Microextraction Gas Chromatography–Mass Spectrometry and Relative Odor Activity Value
Source: Foods. 2023 Dec 18;12(24):4512. doi: 10.3390/foods12244512 (PMC10742727; doi:10.3390/foods12244512)
Supplement: Supplementary file 1 [file foods-12-04512-s001.zip › foods-2719486-supplementary.pdf]

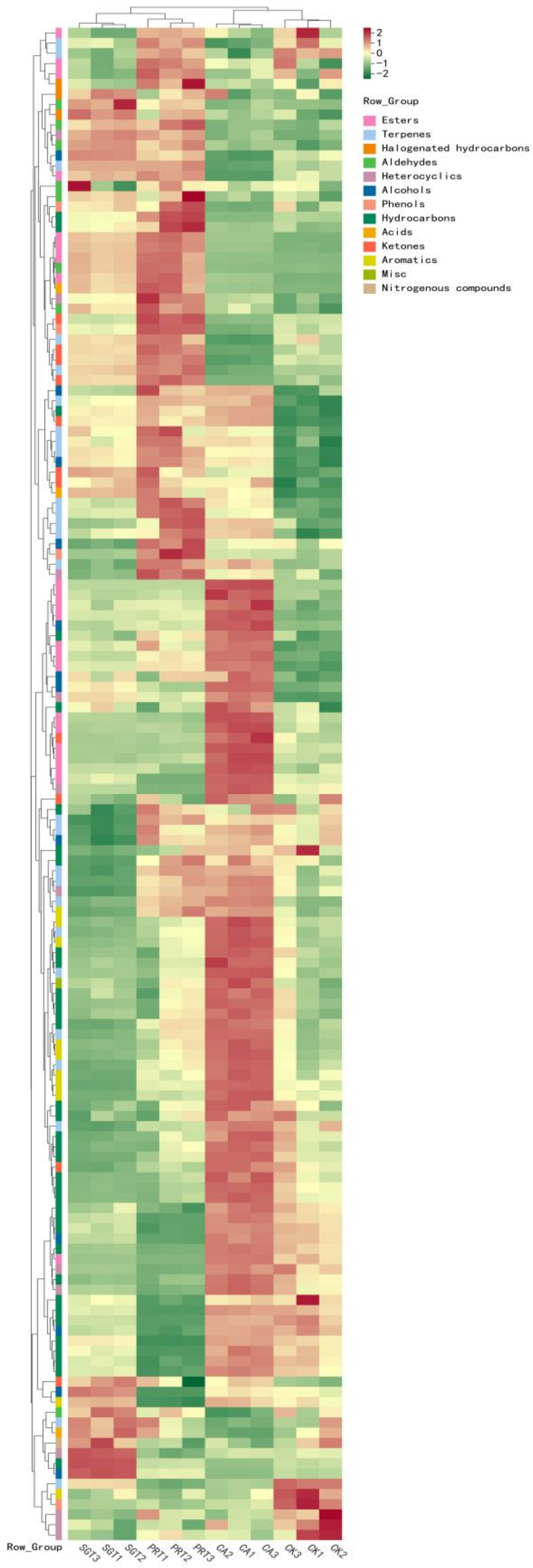

Figure S1. Heat map of volatile compounds, CK refers to fresh tea leaves, CA refers to anaerobic tea leaves, SGT refers to GABA sun-dried green tea and PRT refers to GABA raw Pu-erh tea. Each tea sample was measured in parallel for 3 times.
